# Depletion of phytosterols from intravenous lipid emulsions: to be or not to be

**DOI:** 10.1038/s41390-025-03877-6

**Published:** 2025-02-03

**Authors:** Barath Jagadisan, Anil Dhawan

**Affiliations:** Pediatric Liver GI and Nutrition Centre and Mowat Labs, King’s College Hospital NHS Foundation Trust, London, UK

## Abstract

Long-term total parenteral nutrition (TPN) results in the development of intestinal failure-associated liver disease (IFALD). Fligor et al. have depleted phytosterols from soy-based intravenous lipid emulsions in an attempt to prevent IFALD. Animal studies describe the mechanism of hepatotoxicity of phytosterols, while studies in patients associate high phytosterols with the severity of liver disease. Yet, there are unanswered questions on how much IFALD could be attributed to phytosterols. Challenges to using a phytosterol-depleted ILE include the possibility of the development of oxidation by-products in the lipid, higher cost in the face of as yet unknown clinical benefit from phytosterol depletion and challenges in performing randomised controlled trials to validate the efficacy of newer ILEs.

The outcome of intestinal failure over the years has improved dramatically partly due to improvements in parenteral nutrition formulations. However long-term total parenteral nutrition (TPN) results in the development of intestinal failure-associated liver disease(IFALD) in up to 50% of patients leading to high mortality rates without organ transplantation in those with severe IFALD.^1^ Multiple factors have been attributed to the development of parenteral nutrition-associated liver disease. These include the lack of enteral feeding, high rate and duration of intravenous lipid emulsion (ILE) infusions, excessive glucose infusion, essential fatty acid deficiency, high concentrations of proinflammatory ω-6 polyunsaturated fatty acids, low concentration of the antioxidant α-tocopherol, micronutrient deficiency, disruption in enterohepatic circulation of bile, alteration of gut microbiome, translocation of bacteria from the intestine, intravenous line-associated infections and the presence of phytosterols in lipid emulsion preparations.^2,3^ The study by Fligor et al. in this issue of Paediatric Research attempts to address two of the possible mechanisms of IFALD by studying the low phytosterol lipid emulsion with high tocopherol content.^4^

The composition of lipid emulsions has been the subject of extensive research and newer formulations.^4–12^ Soybean oil-based lipid dimensions (SOLE) provide essential fatty acids but have limitations, including the high content of phytosterols and the predominance of ω-6 fatty acids compared to ω-3 fatty acids. Fish oil-based lipid emulsions (FOLE) have very low levels of phytosterols and high omega-3 fatty acids that can potentially limit inflammation. Their long-term use is limited by their potential to cause essential fatty acid deficiency. In neonates, there are concerns regarding growth and psychomotor development when the arachidonic acid levels do not match or exceed the levels of docosahexaenoic acid levels. This has discouraged using FOLE as the exclusive lipid source in preterm infants.^13^ They are often used presumptively for a few weeks to months to improve liver dysfunction following episodes of sepsis in children receiving TPN.^14^ Although the linoleic acid and alpha-linolenic acid content of FOLE is limited, it has a sufficient quantity of the downstream metabolites, eicosapentaenoic acid and docosahexaenoic acid and also some arachidonic acid that can prevent essential fatty acid deficiency in humans, when dosed at 1 g/kg/day. It can reverse pre-existing essential fatty acid deficiency when dosed at 1.5 g/kg/day.^15^ FOLE has been used in children and adults as the exclusive source of lipids for longer periods in patients with cholestasis in IFALD and is safe without development of essential fatty acid deficiency.^13,16–18^ Recent research has focused on novel oil sources for ILE production with higher ω-3 fatty acids without the potential for essential fatty acid deficiency (Vegaven®).^11,12^ Composite lipid emulsions (CLE) address the limitations of SOLE and FOLE by using a mixed source of oils [*MSF* - 50% medium chain triglycerides (MCT), 40% SO, 10% FO or *SMOF* - 30% SO, 30% MCT, 25% olive oil and 15% FO]. They have been the standard of care for children receiving long-term TPN.^14^ Despite the balance in composition achieved by CLE compared to ILE sourced from single oil sources, the incidence of IFALD while on long-term CLE had been high.^14^ This prompted attempts to study ILE with low phytosterol content since phytosterols have long been suspected to contribute to IFALD.^4,19^

All available ILE except FOLE have high levels of phytosterols. The phytosterol concentration is known to be <10 μg/mL for Omegaven®, 439.1 μg/mL for Intralipid®, 179 ± 10 μg/mL for *SMOF* and 373 ± 12 μg/mL for Vegaven®.^4,11,12^ Since the 1990s, it was observed that infants with IFALD and cholestasis had increased serum phytosterol levels and plant sterols concentrations that were associated with disease severity.^20,21^ An increase in the serum stigmasterol and avenasterol concentrations parallel the portal inflammation and cholestasis during TPN.^8^ The inference of causality of phytosterols to liver disease can be difficult based on this association since cholestasis by itself can increase serum phytosterol levels. The improvement in cholestasis due to IFALD with the use of FOLE leads to the implication of phytosterols in the causation of IFALD, as FOLE has negligible amounts of phytosterols.^22,23^ In children with IFALD in whom SOLE was replaced with FOLE, early changes in serum stigmasterol and IL-8 levels were correlated with later direct bilirubin decline.^10^ Studies that show the effect of phytosterols on bile acid metabolism and also inflammation in the liver in conjunction with endotoxins support the possibility that phytosterols contribute to the pathogenesis of IFALD.^24–26^ The adverse effects of phytosterols in IFALD contrast with the beneficial health effects of enteral phytosterols noted in non-alcoholic fatty liver disease and other disorders. This has been explained by a hypothesis where a distinction is drawn between the presence of free plasma phytosterols when administered through ILE and the chylomicron-packaged phytosterols when enterally administered.^27^ The role of phytosterols in the causation of liver disease can be questioned by the fact that most cases of inherited sitosterolemia do not manifest as liver disease or cholestasis.^28^ The relative contribution of phytosterols to IFALD is debatable, and hence, the benefit that could be accrued by limiting phytosterols in ILE is unpredictable. Irrespective of the primary contribution of phytosterols to the development of IFALD, it is possible the secondary accumulation of phytosterols after the development of a cholestatic form of IFALD could augment liver injury. An earlier study by Guthrie et al. using an ILE with reduced phytosterol content in preterm piglets showed that biochemical markers of cholestasis were lower in those receiving phytosterol-depleted ILE, although the lower survival of piglets and the confounding effect of variable tocopherol levels affected interpretation.^19^

In this issue of the Journal, Fligor et al. depleted phytosterols in SOLE to create a low phytosterol SOLE (L-SOLE) with a total phytosterol content of 74.3 μg/ml and high alpha-tocopherol levels (126 mg/L). Eight-week-old male C57BL/6 J mice were used to create an inflammatory IFALD model. Plasma biomarkers of liver injury (alanine aminotransferase, total bilirubin, bile acids) and histologic liver disease (steatosis, inflammation, F4/80 staining) were studied. L-SOLE was associated with lesser biochemical and histological liver injury.^4^ However, the benefits may not be entirely attributable to low phytosterol content, as the benefit could partly be a result of tocopherol enrichment.^29^ Future studies may need to involve large animal models such as neonatal piglets receiving parenteral nutrition that better reflect human body physiology, metabolism, and the immune system.

Fligor et al. have compared L-SOLE against SOLE as one of the ‘negative’ comparator arms. Since CLE is the standard of care in long-term TPN, the absence of a comparator arm using CLE is intriguing.^4^ Similar studies on novel ILE have used CLE as a comparator arm.^11^ Fligor et al. have demonstrated similar biochemical and histological benefits with L-SOLE and FOLE.^4^ This may tend to suggest that the benefits seen with FOLE may entirely be due to the absence of phytosterol in FOLE rather than due to a predominance of ω-3 fatty acids. It could at least be said that a combination of phytosterol depletion of SOLE and high alpha-tocopherol content can replicate the benefits of FOLE. This will need to be ascertained in the clinical context in children with cholestatic IFALD where L-SOLE needs to be compared against FOLE for use as rescue monotherapy.

Fligor et al. do not discuss the most challenging aspect of depleting phytosterols from SOLE. It is possible that the process of phytosterol depletion could generate oxidation by-products in the ILE, including oxidisation of any remaining phytosterol to generate oxyphytosterols which may have adverse health effects.^30^ These byproducts of production may outweigh any real benefit from phytosterol depletion. The cost of phytosterol depletion is likely to be considerable. Considering the benefits of CLE over SOLE discussed above, any L-SOLE that may come into clinical use will be used as part of a CLE. Phytosterol depletion will add to the cost of existing CLE costs (*SMOF* lipids - $17.73 per 100 mL as of November 2022).^31^ The additional cost will need to be justified by the clinical benefit that is expected from phytosterol depletion.

The demonstration of a clinical benefit of L-SOLE by randomised control trials may be a logistical challenge in terms of the feasibility of trials. A double-blind, randomized controlled trial to compare the safety and efficacy of FOLE compared to SOLE, in reducing the incidence of cholestasis in neonates was terminated early in view of the low incidence of cholestatic IFALD.^32^ Low event rates of cholestatic IFALD with CLE would translate to a need for large sample sizes to demonstrate the superiority of any novel ILE like L-SOLE. This poses challenges to regulatory agencies and also ILE manufacturers.

The authors need to be commended for embarking on a challenging field of research to reduce IFALD in children. Changes in comparator arms and in-depth analysis of possible toxic by-products of phytosterol depletion will shed more light on the relevance and feasibility of this novel product. Novel ILE superior to existing ILE may not be available for clinical use. Until that time, the goal of reducing the frequency of IFALD may need to be realised by understanding and learning to use the existing armamentarium of ILE using novel protocols.
